# Application of an Accelerometric System for Determination of Stiffness during a Hopping Task

**DOI:** 10.1155/2020/3826503

**Published:** 2020-05-21

**Authors:** Artur Struzik, Jerzy Zawadzki, Andrzej Rokita, Bogdan Pietraszewski

**Affiliations:** ^1^Department of Team Sport Games, University School of Physical Education, Wrocław 51-684, Poland; ^2^Department of Biomechanics, University School of Physical Education, Wrocław 51-684, Poland

## Abstract

Currently, there are several computational methods for stiffness during a hopping task, but they do not necessarily yield the same values. Therefore, it is essential that the simplicity of the equipment used does not affect the measurement validity. The aim of this study is to compare the stiffness values during a hopping task recorded in a laboratory environment and those acquired using the Myotest accelerometer. The measurements were performed on a group of 30 untrained female students (age: 23.0 ± 1.7 years, body height: 1.72 ± 0.07 m, and body mass: 64.8 ± 10.0 kg). According to the manual for the Myotest accelerometric system, each study participant performed three sets of 5 hops. Vertical stiffness was determined based on two measurement methods, one using the Myotest accelerometer and the other using Kistler force plates. The mean value (±SD) of vertical stiffness was 19.0 ± 9.3 kN/m in the countermovement phase and 15.1 ± 5.9 kN/m in the take-off phase. Furthermore, the stiffness determined using the Myotest was 30.7 ± 13.3 kN/m. However, significant relationships between the vertical stiffness in the countermovement phase and the Myotest stiffness (*r* = 0.79) and between the vertical stiffness in the take-off phase and the Myotest stiffness (*r* = 0.89) were found. The relationships between the vertical stiffness (in the countermovement and take-off phases) and the stiffness estimated using the Myotest allow us to conclude that despite the significantly overestimated stiffness value, the Myotest accelerometer can still be used for determination of the stiffness trends, e.g., following training. The overestimated stiffness values can result both from inaccuracy in the determination of ground contact time and flight time by the Myotest accelerometer and from the use of an equation that assumes that the movement of the center of mass has a harmonic profile.

## 1. Introduction

Evaluation and monitoring of biomechanical variables have become an important element in the quantitative analysis of athletic performance. Sports coaches will obtain valuable information from measurements carried out under conditions as close as possible to those during competitions. Therefore, they are often skeptical of analyses performed under isolated laboratory conditions. However, recent technological innovations related to the miniaturization of wearable sensors that do not influence the technical movements of athletes allow movement analysis to be performed during sporting activities. An example of a tool that allows the measurement of acceleration during motion and under training conditions is the Myotest performance measuring system (Myotest SA, Sion, Switzerland).

The Myotest accelerometric system is a wireless handheld device weighing just a few ounces (59 g) and is attached to a specially designed belt at the pelvic level. This 3-D accelerometer allows the estimation of variables such as jump height, time of contact, reactivity, and stiffness during a hopping task. Time of contact refers to time when the feet (at least one) are in contact with the ground between the flight phases. Reactivity should be understood as the reactive strength index (RSI), i.e., as the ratio of jump height to contact time [1]. We can also find in the Myotest guide that “*muscular rigidity*, *which is usually called stiffness*, *is an interesting indicator enabling you to find the ideal muscular tension for bouncing in running events or team sports*, *for instance*.” However, the problem of the “stiffness” estimated by the Myotest seems more complex than this definition.

Stiffness is a quantitative measure of the elastic properties of the body and is expressed as a ratio of the deforming force to the deformation length (most commonly in relation to longitudinal deformation) [2]. Therefore, stiffness represents the measure of resistance to strain and is described as an essential factor in the optimization of human locomotion [3–5]. Dalleau et al. [6] argued that stiffness is also related to the maximal performance of single and cyclic movements. When hopping, the human body (movement of the center of mass) resembles a bouncing ball. Therefore, the term “*bouncing gait*” has been used to describe the human body during hopping tasks where lower limbs perform the function of “*springs*” responsible for center of mass (COM) movements [4, 5, 7]. Therefore, a hopping human body can be modeled by using a simple spring-mass model that contains a single (linear and massless) “*leg spring*” and a point that represents the total body mass [3]. Leg stiffness (defined as the ratio of changes in the ground reaction force to the respective changes in “*spring length*” representing both lower limbs) and vertical stiffness (defined as the ratio of changes in the ground reaction force to the respective vertical displacement of the COM) are commonly used to describe the mechanical properties of a “*spring*” representing the lower limbs during a hopping task [8].

The Myotest guide does not give an unambiguous answer as to which of the above types of stiffness (leg or vertical) is the value provided by the Myotest accelerometric system during the hopping test. Some authors equate the stiffness value estimated by the Myotest with leg stiffness [9–12]. However, the accelerometer is not capable of measuring the change in “*spring length*.” Moreover, estimations of stiffness using an accelerometer also do not provide insights about the ratios of stiffness in individual joints. Therefore, it should be assumed that the stiffness value estimated by the Myotest is vertical stiffness. The Myotest accelerometric system is recognized as a reliable and valid tool for the estimation (despite significant overestimation) of jump height based on the flight time method [13–17]. However, it seems that the problem of the stiffness determined using an accelerometer is currently not properly investigated. To our knowledge, only a few studies [9, 10] have raised the issue of stiffness estimated by the Myotest.

In the process of sports training control, it is necessary to quantify the effects of the exercises and loads applied. Therefore, the use of a portable measuring device is a compromise between measurements under laboratory conditions and those under training conditions. However, there are currently several computational methods for (vertical) stiffness, but they do not necessarily yield the same values [8, 18–20]. Therefore, it is essential that the simplicity of the equipment used does not affect the measurement validity. The aim of this study is to compare the stiffness values during a hopping task recorded in a laboratory environment and those acquired using the Myotest accelerometer.

## 2. Materials and Methods

The measurements were performed on a group of 30 untrained female students from the University School of Physical Education. They were persons with no competitive-level sports training (within a period of at least 5 years before the experiment) and with no injuries to the musculoskeletal (motion) system. The study group was characterized by the following mean parameters (±SD): body height: 1.72 ± 0.07 m, body mass: 64.8 ± 10.0 kg, and age: 23.0 ± 1.7 years. The tests were carried out in the Biomechanical Analysis Laboratory (with PN-EN ISO 9001: 2009 certification). Each subject completed all trials in the same time period of test days (in the morning) to eliminate any influence of circadian variation. Subjects refrained from physical activity for 24 hours before testing, to avoid any interference in the experiment. Prior to the measurements, the participants were familiarized with the purpose of the study and gave written consent for participation in the experiment. Before the test, the subjects were informed of the activities they were supposed to perform and were motivated to properly perform the task. The research project was approved by the Senate's Research Bioethics Commission, and the procedure complied with the Declaration of Helsinki regarding human experimentation. We followed the methods of Struzik and Pietraszewski [21].

Each study participant performed three sets of 5 hops (hopping test). The measurement procedure was conducted in accordance with the Myotest performance measuring system: quick start guide (jump–plyometry test). The trials were simultaneously recorded by the Myotest accelerometric system (Myotest SA, Sion, Switzerland) and by two force plates (9286A, Kistler Group, Winterthur, Switzerland). The sampling frequencies of the signal from the force plates and the accelerometric system were set at 500 Hz. This sampling frequency is the maximum common value for both systems. The use of force plates is usually considered the gold standard [13, 15].

**Figure 1:**
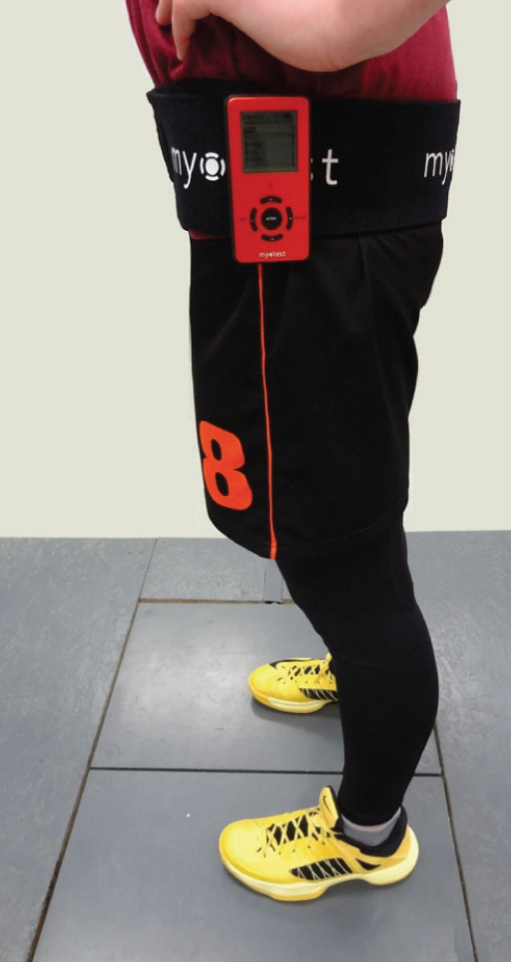
One of the participants standing on the force plates with the belt to which the Myotest accelerometer is attached vertically on the left side of the body at the pelvic level.

Prior to the measurements, a 10-minute-long warm-up, which included jogging (shuttle runs over a distance of 10 m, at a moderate pace of ca. 10 sections per minute), a series of hops, and a familiarization test task, was administered. Each study participant started performing a trial series after becoming familiar with the test. After the trial series, the proper research procedure began. Next, the participant was asked to perform a series of 5 bilateral hops (3 sets) from the standing position to the maximum height (performed as a bounce action on the fore foot) and with minimal time of contact with the ground. The whole part of the hopping test took place on a rigid surface (force plates). As indicated by the guide, the participant wore a belt with the Myotest accelerometer attached vertically on the left side of the body at the pelvic level (fastened around both greater trochanters of the femurs and the medium part of the gluteal region). Before each trial, the subjects were asked to stand over the force plates (each foot on a separate plate) while assuming a vertical posture with arms akimbo, looking straight ahead and standing still (Figure 1). The hopping test instructions given were as follows (according to Myotest guide): “*at the short beep from accelerometer*, *perform a countermovement jump*, *then bounce back up five times as high as possible and with a ground contact time that is as short as possible*, *while keeping your hands on your waist* (*jump off the soles of the foot with minimal bending of the knees*, *like on a trampoline*).” After 5 hops, the participant reassumed a vertical standing posture, and the double beep from the accelerometer signals the end of the test. During the experiment, the participant was asked to rest her palms on her hips to exclude the effect of arm swing on hopping performance. Landings were performed on the same plates as take-offs. According to the Myotest guide, a one-minute rest took place between test repetitions. Errors in hopping task execution are signaled by a deep beep from the accelerometer. The Myotest accelerometric system tolerates two errors before automatically stopping the test. An error message is generated if the following points are not observed (according to Myotest guide): “(1) *execute the movements energetically so that the Myotest can clearly detect them*, (2) *stand still before the starting beep*, (3) *ground contact time must be short and clearly below the time of flight*, *and* (4) *perform a total of 5 bounces*.” During performance of the hopping test, the participant should take-off with the knees and ankles extended and land in a similarly extended position. The test was repeated if the lower limbs were flexed at the knee and/or hip joints during the flight phase (incorrectly performed hopping task).

**Figure 2:**
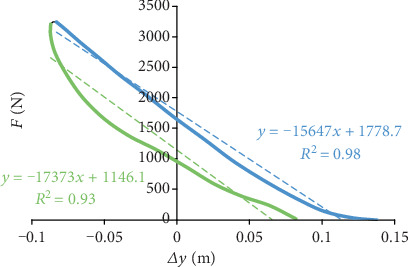
Ground reaction force depending on the COM vertical displacement for one of the study participants during the hopping test (for one of the hops), along with trend lines and equations that describe these dependences for the parts of the countermovement (marked green) and take-off phases (marked blue) and the values of coefficients of determination *R*^2^.

Further analysis focused on the attempt with the highest mean height of hops obtained by each participant. From the hopping task, 5 hops were analyzed without taking into account the starting countermovement jump. The values of all presented variables were averaged for the five analyzed hops to obtain results analogous to those obtained from the Myotest. The hopping test was used with some simplifications that resulted from the use of the spring-mass model, which characterizes both running and hopping. The model assumes that the human body consists of a material point representing the total mass of the body; a massless “spring” representing both lower limbs, which performs the supporting function; and a parallel source of force resulting from the active action of the muscles involved in the take-off [3]. Based on the vertical ground reaction force (*F*) recorded by the force plates (the ground reaction forces registered by both force plates were added up), it was possible to determine the flight time (*t*_f_) and ground contact time (*t*_c_) during the hopping task. The instantaneous pattern of changes in the height of the COM (*y*) was calculated by double integration of the COM vertical acceleration, as calculated from the vertical ground reaction force [4]. The vertical (quasi-) stiffness (*K*_v_ = Δ*F*/Δ*y*) of the human body during the hopping task was determined as the ratio of the change in the ground reaction force (Δ*F*) to the corresponding change in the height of the COM (Δ*y*) separately for the countermovement and take-off phases, similar to the method described by Struzik and Zawadzki [22]. To reliably estimate vertical stiffness, it is necessary to determine the relationship *F*(Δ*y*) shown in Figure 2. The slope coefficient for part of the curve *F*(Δ*y*) equals the numerical value of stiffness in this range. Vertical stiffness was calculated for the parts of the countermovement and take-off phases where the slope of the *F* curve with respect to the Δ*y* axis was relatively constant and the *F*(Δ*y*) profile was nearly linear. For the countermovement phase (marked green in Figure 2), this range was the part between the moment of landing on the plates and the lowest location of the COM (Δ*y*_max_). The boundaries of the part for the take-off phase (marked blue in Figure 2) were represented by the local maximum of the ground reaction forces (point *F*_max_ from which ground reaction forces decreased only) and the moment of take-off from the plates [22]. This observation holds true only if the value of the coefficient of determination *R*^2^ that expresses the quality of adjustment of the trend line to the relevant part of the *F*(Δ*y*) curve is sufficiently high (over 0.6) [23]. If the points Δ*y*_max_ and *F*_max_ occur at exactly the same time, then the whole *F*(Δ*y*) curve is analyzed. If not, then the part of the *F*(Δ*y*) curve between the Δ*y*_max_ and *F*_max_ points (marked in black in Figure 2) is omitted to maintain the maximum possible linearity of the studied parts of the countermovement and take-off phases. It is possible that the *F*(Δ*y*) curve intersects [7], for example, in the upper part, as shown by Choukou et al. [9], which causes the *F*_max_ point to appear before the Δ*y*_max_ point. Then, the profile of the *F*(Δ*y*) curve should be considered individually, and the boundary of the analyzed parts of the countermovement and take-off phases should be modified. For example, the analyzed countermovement phase part will end at the point *F*_max_, and the analyzed part of the take-off phase will begin at the point Δ*y*_max_.

The Myotest accelerometer was used to record the following variables during the hopping test: jump height (*h*_Myo_), ground contact time (*t*_c‐Myo_), and stiffness (*K*_Myo_). In the Myotest guide, the manufacturer did not explain how the values of individual variables are estimated. However, based on the accelerometer capabilities, one can guess that the values of jumping height (*h*_Myo_) and ground contact time (*t*_c‐Myo_) were determined based on the duration of the flight and ground contact phases [9]. Based on the jump height (*h*_Myo_) recorded by the Myotest accelerometer, the flight time (*t*_f‐Myo_) could be determined using the following formula:

(1) $$\begin{array}{c} t_{\text{f}‐\text{Myo}}=2\cdot \sqrt{\frac{2\cdot h_{\text{Myo}}}{g}}, \end{array}$$

where *g* is the acceleration due to gravity [24]. Furthermore, (vertical quasi-) stiffness (*K*_v‐Myo_) can be evaluated using the equation described by Dalleau et al. [6], which assumes that the curve reflecting the ground reaction force versus time is a part of the sine wave:

(2) $$\begin{array}{c} K_{\text{v}‐\text{Myo}}=\frac{m\cdot \pi \cdot \left(t_{\text{f}‐\text{Myo}}+t_{\text{c}‐\text{Myo}}\right)}{{t_{\text{c}‐\text{Myo}}}^{2}\cdot \left(\left(\left(t_{\text{f}‐\text{Myo}}+t_{\text{c}‐\text{Myo}}\right)/\pi \right)-\left(t_{\text{c}‐\text{Myo}}/4\right)\right)}, \end{array}$$

where *K*_v‐Myo_ is the vertical stiffness, *m* is the body mass, *t*_f‐Myo_ is the flight time, and *t*_c‐Myo_ is the ground contact time. Therefore, it may be accepted that the stiffness value estimated by the Myotest is the vertical (quasi-) stiffness.

The sample size was determined based on the power analysis. For *n* = 30, the power of applied statistical tests (1 − *β*) is close or equal 1. The Shapiro-Wilk (*W*) and Lilliefors tests were used to examine the distribution of individual variables. All the studied variables had a distribution close to normal. Therefore, parametric tests were used for further analyses. Pearson's *r* correlation coefficient was used to evaluate the concurrent validity of the Myotest accelerometric system and force plate. The significance of the correlation coefficient value was verified with the *t*-test. To demonstrate possible differences between the values of the variables obtained from different measuring devices, Student's *t*-test of significance of differences for dependent variables was used. In all tests performed, the level of significance was set at *α* = 0.05. Statistical calculations were made by means of the Statistica 13.3 software package (TIBCO Software Inc., Palo Alto, CA). Furthermore, the remaining calculations were made using a Microsoft Excel 2016 spreadsheet (Microsoft Corporation, Redmond, WA). Additionally, concurrent validity was analyzed through a Hopkins [25] spreadsheet to quantify the relationship between the practical (Myotest) and criterion (force plate) measures. The validity spreadsheet is based on simple linear regression to derive a calibration equation, a typical error of the estimate, and Pearson's *r* correlation coefficient. The criterion was the dependent variable, and the practical was the predictor in a consecutive pairwise manner. The typical error of the estimate was standardized (SEE) by dividing by the SD of the criterion. SEE was evaluated using half the thresholds of the modified Cohen scale: <0.1, trivial; 0.1–0.3, small; 0.3–0.6, moderate; 0.6-1.0, large; 1.0-2.0, very large; and >2.0, extremely large. Uncertainty in the estimates was expressed as 90% confidence limits. To complement the correlation analysis, Bland-Altman plots were used to visualize the mean of the difference (bias) and the limits of agreement (95% confidence intervals).

## 3. Results

**Figure 3:**
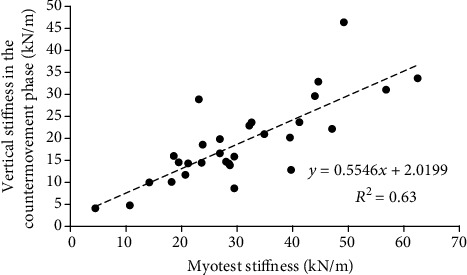
Vertical stiffness in the countermovement phase versus stiffness determined using the Myotest accelerometric system during the hopping test with an equation describing the trend line and the value of the determination coefficient *R*^2^.

**Figure 4:**
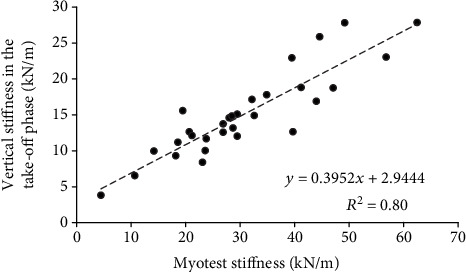
Vertical stiffness in the take-off phase versus stiffness determined using the Myotest accelerometric system during the hopping test with an equation describing the trend line and the value of the determination coefficient *R*^2^.

The mean value (±SD) of vertical stiffness was 19.0 ± 9.3 kN/m in the countermovement phase and 15.1 ± 5.9 kN/m in the take-off phase during the hopping test. Furthermore, the stiffness determined using the Myotest accelerometric system was 30.7 ± 13.3 kN/m. Therefore, the stiffness values determined using the Myotest were significantly higher than the stiffness values determined using the force plate in both the countermovement (Δ = 11.7 ± 8.2 kN/m) and take-off phases (Δ = 15.6 ± 8.5 kN/m). However, significant relationships between the vertical stiffness in the countermovement phase and the Myotest stiffness (*r* = 0.79, SEE = 0.77, Figure 3) and between the vertical stiffness in the take-off phase and the Myotest stiffness (*r* = 0.89, SEE = 0.50, Figure 4) were found. A significant difference between the vertical stiffness values in the countermovement phase and those in the take-off phase (Δ = 4.0 ± 5.7 kN/m, *p* < 0.001) was also found.

**Figure 5:**
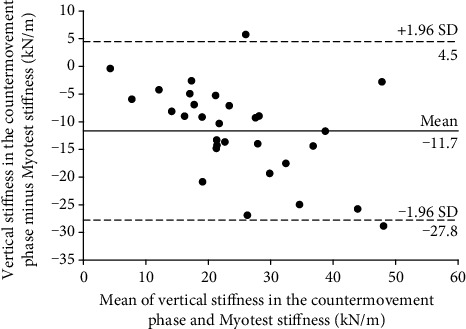
Bland-Altman plot of the force plate (in the countermovement phase) and Myotest stiffness (95% limit of agreement is 16.1 kN/m).

**Figure 6:**
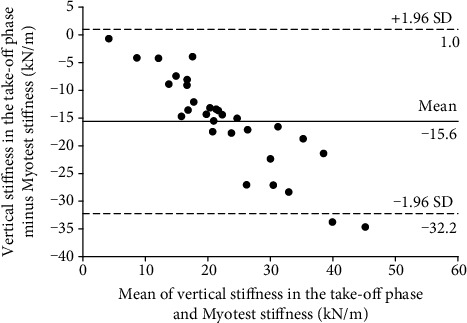
Bland-Altman plot of the force plate (in the take-off phase) and Myotest stiffness (95% limit of agreement is 16.6 kN/m).

Bland-Altman plots are presented in Figures 5 and 6. For any measurement system to be valid, most of the paired differences should lie within the 95% limits of agreement, whereas their mean can help identify whether any system underestimates or overestimates measurements relative to the criterion (bias). The results indicate that the Myotest accelerometric system overestimated measurements of stiffness during the hopping test. In Figures 5 and 6, 28 of the 30 analyzed measurements are within the limits of agreement. However, significant relationships between the paired differences and means were found, indicating that the bias is not constant over the entire range. Therefore, as the Myotest stiffness value increases, the vertical stiffness estimation error in the countermovement (*r* = −0.51) and take-off phases (*r* = −0.90) also increases.

Table 1 contains the mean values (±SD) of ground contact time and flight time obtained with the Myotest accelerometric system and force plate. The ground contact time estimated by the Myotest was significantly shorter than that obtained from the force plate measurements. In turn, the flight time estimated by the Myotest was significantly longer than that obtained from the force plate measurements.

Moreover, the value of stiffness determined using the force plate time measurements (*t*_c_ and *t*_f_) and the equation described by Dalleau et al. [6] was *K*_D_ = 15.6 ± 5.7 kN/m. *K*_D_ was significantly lower than the vertical stiffness in the countermovement phase (Δ = 3.4 ± 5.2 kN/m, *p* < 0.01) and the Myotest stiffness (Δ = 15.1 ± 8.4 kN/m) and was at a similar level as the vertical stiffness in the take-off phase.

## 4. Discussion

Although popular motor ability tests can be performed in a simple manner and under any conditions (for example, the Sargent vertical jump test), modern measurement equipment provides more accurate information about a particular ability or variable. The development of technology also allows a more objective and precise evaluation. It is also becoming easier to collect more data than was previously possible using conventional methods and tools. Therefore, it is fundamental that coaches should utilize available methods for applying scientific output in sports training. Utilizing these methods is likely to provide them with feedback on the current skill level of an athlete and the efficiency of the particular practice stimuli used and will help them plan future training programs. Modern measurement tools also offer possibilities for the detection of irregularities in an athlete's body that might lead to injuries.

Reliability can be defined as the consistency of measurements (test-retest) [13]. Choukou et al. [9] and Ruggiero et al. [10] stated that the stiffness values estimated using the Myotest accelerometer showed a high level of reliability. On the other hand, validity refers to the ability of a measurement tool to reflect what it is designed to measure [13]. However, the problem of validity of stiffness measurements using Myotest is much more complex and not yet fully explained. Both the laboratory and field tests must be valid and reliable in order to properly use information obtained on their basis. Therefore, laboratory measuring systems [26–28], portable measuring tools [29–31], calculation methods, and measuring movements [8, 20, 24, 32–34] are subject to verification. Compared to other devices for field-based jumping evaluation, the Myotest has the advantages of being small and portable, easy to handle, relatively cheap, able to provide immediate results, and usable on particular surfaces (e.g., on the sand), which allows measurements under any conditions without limitations on the measurement space [13, 14]. However, it cannot be used during a game or competition [35].

The stiffness determined using the Myotest accelerometric system during the hopping test was significantly higher than the vertical stiffness determined using the force plate measurements in the countermovement and take-off phases. Therefore, the Myotest overestimated measurements of stiffness, as in other studies [9, 10]. Choukou et al. [9] noted significantly higher values of stiffness estimated by the Myotest (by 7.8 kN/m) during the hopping test than the vertical stiffness values determined using a force plate. The stiffness estimation method during hopping presented by Dalleau et al. [6] assumed that the curve that describes the dependence of the ground reaction force on time is a part of the sine wave and therefore that the COM motion is harmonic. However, this method is only the first half of oscillations, as a result of which it does not strictly meet the assumptions of harmonic motion. The description (equation) is appropriate for the steady course of such oscillations. Notably, the method presented by Dalleau et al. [6] can cause the values of vertical stiffness to be significantly overestimated, especially at relatively low hopping frequencies. Based on the given hopping test instruction and the obtained *t*_c_ and *t*_f_ values, it can be concluded that the hopping frequency chosen by the participants in this study was low.

On the other hand, Hobara et al. [20] reported that the stiffness estimation method presented by Dalleau et al. [6] significantly underestimates vertical stiffness values during hopping compared to those obtained from other calculation methods. However, Hobara et al. [20] took all measurements on a force plate without using the accelerometer. In this study, the values of stiffness determined using the force plate measurements (*t*_c_ and *t*_f_) and the equation described by Dalleau et al. [6] were also significantly lower than the vertical stiffness values in the countermovement phase and the Myotest stiffness values and were at a similar level as the vertical stiffness values in the take-off phase. The overestimated stiffness value by the Myotest accelerometer during the hopping test can therefore result from inaccuracy in the determination of ground contact time and flight time. These two variables are mainly responsible for the stiffness value estimated using the equation presented by Dalleau et al. [6]. In this study, the ground contact time estimated by the Myotest was significantly shorter than that obtained from the force plate measurements. In turn, the flight time estimated by the Myotest was significantly longer than that obtained from the force plate measurements. The trends in the mentioned differences coincide with those presented by other authors [9, 13]. Choukou et al. [9] stated that the measurement of ground contact time by the Myotest during the hopping test is nonvalid. The most accurate devices for recording vertical jump flight time and ground contact time are force plates, which allow precise identification of the instant of take-off (the point at which the feet lose contact with the ground and the value of vertical ground reaction force drops to zero) and instant of landing (the feet land in the same position as take-off). It is assumed that the COM height at take-off is relatively the same as that at landing [24]. The Myotest estimates flight time using the time difference between the positive (during take-off phase) and negative (during landing phase) peaks of vertical velocity. However, the maximal positive vertical velocity is reached shortly before the instant of take-off, and the maximal negative vertical velocity is reached shortly after the instant of landing. Therefore, the flight time recorded by the Myotest accelerometer is overestimated, and the ground contact time is underestimated [9, 13, 24]. The ground contact time and flight time values presented in Table 1 confirm the above assumptions, which can significantly distort the stiffness values estimated by the Myotest during hopping.

A significant relationship between the vertical stiffness in the countermovement phase and the Myotest stiffness obtained during hopping was found. This relationship was very high but also had a large SEE. A significant relationship between the vertical stiffness in the take-off phase and the Myotest stiffness was also found. This relationship was very high and had a moderate SEE. When the SEE is large, the predicted *y* values are scattered widely above and below the regression line (Figures 3 and 4). However, based on the Bland-Altman plots (Figures 5 and 6), most of the paired differences are within the 95% limits of agreement. Therefore, it can be concluded that the Myotest accelerometric system is valid but overestimates the vertical stiffness values during hopping. Moreover, greater overestimation is observed with an increase in the criterion value. Therefore, the Myotest stiffness is not interchangeable with respect to the values obtained from other measurement devices and methods. The Myotest accelerometric system determines an approximate value that can provide information about only changes in vertical stiffness during the hopping test.

Determination of the vertical stiffness during the hopping task requires several assumptions that sometimes seem to have been omitted, whereas measurement validity would require verification of these assumptions. The simplest case is when *F*_max_ occurs exactly at the same time as Δ*y*_max_. Without this synchronization, it would be necessary to determine which of these events occur first and, consequently, to modify the equation to reproduce the profile of the *F*(Δ*l*) curve as accurately as possible. The increase in the ground reaction force with respect to the COM displacement should be linear or close to linear over the whole duration of the contact with the ground phase. If the moment of occurrence of *F*_max_ divided *t*_c_ into two halves (harmonic movement), it would theoretically mean the same values of vertical stiffness during the countermovement and take-off phases. Meeting the above conditions would justify using one value as vertical stiffness for a specific movement while neglecting the calculations for the take-off phase [36]. Ferris and Farley [4] emphasized that during hopping, *F*_max_ and Δ*y*_max_ do not necessarily occur at the same time. It is assumed that for a hopping frequency lower than 2 Hz, lower limbs stop behaving as linear springs, thereby distorting the *F*(Δ*l*) profile [7, 37]. In this work, the vertical stiffness values in the countermovement phase were significantly higher than those in the take-off phase. Therefore, to fully understand the phenomena occurring during human motion, it seems necessary to determine the vertical stiffness for both phases of motion separately. The assumption that the value of vertical stiffness in the countermovement phase is always the same as that in the take-off phase may be too much of a simplification. Luhtanen and Komi [38] estimated vertical stiffness during running and long jump with a division into the eccentric and concentric phases. Furthermore, the stiffness determined based on observation during motion should be viewed as quasi-stiffness, i.e., the ability of the human body to resist external displacements while ignoring the temporal profile of the displacement. Vertical stiffness is not stiffness viewed in strict terms due to the substantial contribution of other factors (such as damping and inertia) that affect the *F*(Δ*y*) relationship, especially during transient states [2].

Despite the clearly established procedures for hopping test performance and Myotest accelerometer fixation, between-subject differences in hopping technique (differences in jumping technique due to gender [23, 36, 37, 39] and sports training [36, 40–42]), elastic belt fastening and positioning around the hips, and, consequently, Myotest orientation may cause unexpected device displacements during hopping. Because the Myotest was applied vertically to an elastic belt, the accelerometer may have moved forward a certain amount during the countermovement or take-off phase due to trunk flexion. This movement would have caused vertical acceleration, and consequently, the vertical velocity and time (ground contact and flight) recordings would present a certain amount of random error [13]. It seems that stable fixation on the dorsal portion of the pelvic girdle of the jumping person can provide less sensitivity to undesirable accelerometer movement [30, 43, 44]. As a result, Castagna et al. [15] and Choukou et al. [9] decided to place the Myotest accelerometer in such a way.

A certain limitation of this study can be the studied group of untrained female. Based on other studies [36], it can be expected that the absolute stiffness value will be higher for male than for female and higher for athletes than for untrained people. Therefore, according to the relationships presented in this paper (between the paired differences and means), even larger bias values (larger overestimation of vertical stiffness values by the Myotest) can be expected in groups of males and athletes.

## 5. Conclusions

The relationships between vertical stiffness (in the countermovement and take-off phases) and the stiffness estimated using the Myotest accelerometric system allow us to conclude that, despite the significantly overestimated value of stiffness, the Myotest accelerometer can be used for determination of the stiffness trend. Therefore, this measurement device offers only an approximate stiffness value that can provide information about changes, e.g., following training. Therefore, the Myotest stiffness is not interchangeable with respect to the values obtained from other measurement devices and methods because of systematic overestimation. The overestimated stiffness value can result both from inaccuracy in the determination of ground contact time and flight time by the Myotest accelerometer and from the use of an equation that assumes that the movement of the center of mass has a harmonic profile.

## Figures and Tables

**Table 1:** Mean values (±SD) of ground contact time (*t*_c_) and flight time (*t*_f_) obtained with the Myotest accelerometric system and force plates.

	*t*_c_ (s)	*t*_f_ (s)	*t*_c_ + *t*_f_ (s)
Myotest	0.18 ± 0.06	0.51 ± 0.03	0.68 ± 0.07
Force plate	0.25 ± 0.07	0.44 ± 0.04	0.69 ± 0.07
Δ	−0.08 ± 0.01^∗^	0.07 ± 0.02^∗^	−0.01 ± 0.01

Δ represents differences between the values of times obtained with the Myotest accelerometric system and force plates. ^∗^Statistically significant at *p* < 0.05.

## Data Availability

The data used to support the findings of this study are available from the corresponding author upon request.

## References

[1] Mcclymont D., Hore A. (2003). *Use of the reactive strength index RSI as a plyometric monitoring tool*.

[2] Latash M. L., Zatsiorsky V. (2016). *Biomechanics and Motor Control: Defining Central Concepts*.

[3] Blickhan R. (1989). The spring-mass model for running and hopping. *Journal of Biomechanics*.

[4] Ferris D. P., Farley C. T. (1997). Interaction of leg stiffness and surface stiffness during human hopping. *Journal of Applied Physiology*.

[5] Farley C. T., Houdijk H. H., van Strien C., Louie M. (1998). Mechanism of leg stiffness adjustment for hopping on surfaces of different stiffnesses. *Journal of Applied Physiology*.

[6] Dalleau G., Belli A., Viale F., Lacour J. R., Bourdin M. (2004). A simple method for field measurements of leg stiffness in hopping. *International Journal of Sports Medicine*.

[7] Farley C. T., Blickhan R., Saito J., Taylor C. R. (1991). Hopping frequency in humans: a test of how springs set stride frequency in bouncing gaits. *Journal of Applied Physiology*.

[8] Serpell B., Ball N., Scarvell J., Smith P. (2012). A review of models of vertical, leg, and knee stiffness in adults for running, jumping or hopping tasks. *Journal of Sports Sciences*.

[9] Choukou M. A., Laffaye G., Taiar R. (2014). Reliability and validity of an accelerometric system for assessing vertical jumping performance. *Biology of Sport*.

[10] Ruggiero L., Dewhurst S., Bampouras T. M. (2016). Validity and reliability of two field-based leg stiffness devices: implications for practical use. *Journal of Applied Biomechanics*.

[11] Hobara H., Hashizume S., Kobayashi Y. (2017). Effects of prophylactic ankle and knee braces on leg stiffness during hopping. *Open Access Journal of Sports Medicine*.

[12] Kurt C., Kafkas M. E., Kurtdere İ., Selalmaz O. (2018). Influence of traditional and cluster set plyometric warm-ups on reactive strength index and leg stiffness in male rugby players. *Isokinetics and Exercise Science*.

[13] Casartelli N., Muller R., Maffiuletti N. A. (2010). Validity and reliability of the myotest accelerometric system for the assessment of vertical jump height. *The Journal of Strength & Conditioning Research*.

[14] Nuzzo J. L., Anning J. H., Scharfenberg J. M. (2011). The reliability of three devices used for measuring vertical jump height. *The Journal of Strength & Conditioning Research*.

[15] Castagna C., Ganzetti M., Ditroilo M., Giovannelli M., Rocchetti A., Manzi V. (2013). Concurrent validity of vertical jump performance assessment systems. *The Journal of Strength & Conditioning Research*.

[16] Hojka V., Tufano J. J., Maly T. (2018). Concurrent validity of Myotest for assessing explosive strength indicators in countermovement jump. *Acta Gymnica*.

[17] Stanton R., Doering T. M., Macgregor C., Borges N., Delvecchio L. (2018). Validity of a contact mat and accelerometric system to assess countermovement jump from flight time. *Measurement in Physical Education and Exercise Science*.

[18] Butler R. J., Crowell H. P., Davis I. M. (2003). Lower extremity stiffness: implications for performance and injury. *Clinical Biomechanics*.

[19] Brughelli M., Cronin J. (2008). A review of research on the mechanical stiffness in running and jumping: methodology and implications. *Scandinavian Journal of Medicine & Science in Sports*.

[20] Hobara H., Inoue K., Kobayashi Y., Ogata T. (2014). A comparison of computation methods for leg stiffness during hopping. *Journal of Biomechanics*.

[21] Struzik A., Pietraszewski B. (2019). Relationships between Hamstrings-to-Quadriceps Ratio and Variables Describing Countermovement and Drop Jumps. *Applied Bionics and Biomechanics*.

[22] Struzik A., Zawadzki J. (2016). Application of force-length curve for determination of leg stiffness during a vertical jump. *Acta of Bioengineering and Biomechanics*.

[23] Granata K. P., Padua D. A., Wilson S. E. (2002). Gender differences in active musculoskeletal stiffness. Part II. Quantification of leg stiffness during functional hopping tasks. *Journal of Electromyography and Kinesiology*.

[24] Linthorne N. P. (2001). Analysis of standing vertical jumps using a force platform. *American Journal of Physics*.

[25] Hopkins W. G. (2015). Spreadsheets for analysis of validity and reliability. *Sportscience*.

[26] Windolf M., Gotzen N., Morlock M. (2008). Systematic accuracy and precision analysis of video motion capturing systems—exemplified on the Vicon-460 system. *Journal of Biomechanics*.

[27] Zawadzki J., Bober T., Siemieński A. (2010). Validity analysis of the biodex system 3 dynamometer under static and isokinetic conditions. *Acta of Bioengineering and Biomechanics*.

[28] Słomka K. J., Sobota G., Skowronek T., Rzepko M., Czarny W., Juras G. (2017). Evaluation of reliability and concurrent validity of two optoelectric systems used for recording maximum vertical jumping performance versus the gold standard. *Acta of Bioengineering and Biomechanics*.

[29] Pueo B., Jimenez-Olmedo J. M., Lipinska P., Busko K., Penichet-Tomas A. (2018). Concurrent validity and reliability of proprietary and open-source jump mat systems for the assessment of vertical jumps in sport sciences. *Acta of Bioengineering and Biomechanics*.

[30] Staniak Z., Buśko K., Górski M., Pastuszak A. (2018). Accelerometer profile of motion of the pelvic girdle in butterfly swimming. *Acta of Bioengineering and Biomechanics*.

[31] Cai L., Ma Y., Xiong S., Zhang Y. (2019). Validity and reliability of upper limb functional assessment using the microsoft kinect V2 sensor. *Applied Bionics and Biomechanics*.

[32] Hatze H. (1998). Validity and reliability of methods for testing vertical jumping performance. *Journal of Applied Biomechanics*.

[33] Kibele A. (1998). Possibilities and limitations in the biomechanical analysis of countermovement jumps: a methodological study. *Journal of Applied Biomechanics*.

[34] Slomka K., Juras G., Sobota G., Bacik B. (2013). The reliability of a rambling–trembling analysis of center of pressure measures. *Gait & Posture*.

[35] Roell M., Mahler H., Lienhard J., Gehring D., Gollhofer A., Roecker K. (2019). Validation of wearable sensors during team sport-specific movements in indoor environments. *Sensors*.

[36] Struzik A. (2018). Leg stiffness during vertical jumps to maximal and specific heights. *Studia i Monografie Akademii Wychowania Fizycznego we Wrocławiu, no. 128*.

[37] Hobara H., Kato E., Kobayashi Y., Ogata T. (2012). Sex differences in relationship between passive ankle stiffness and leg stiffness during hopping. *Journal of Biomechanics*.

[38] Luhtanen P., Komi P. V. (1980). Force-, power-, and elasticity-velocity relationships in walking, running, and jumping. *European Journal of Applied Physiology and Occupational Physiology*.

[39] Padua D. A., Carcia C. R., Arnold B. L., Granata K. P. (2005). Gender differences in leg stiffness and stiffness recruitment strategy during two-legged hopping. *Journal of Motor Behavior*.

[40] di Cagno A., Baldari C., Battaglia C. (2009). Factors influencing performance of competitive and amateur rhythmic gymnastics—gender differences. *Journal of Science and Medicine in Sport*.

[41] Hobara H., Kimura K., Omuro K. (2010). Differences in lower extremity stiffness between endurance-trained athletes and untrained subjects. *Journal of Science and Medicine in Sport*.

[42] Ambegaonkar J. P., Shultz S. J., Perrin D. H., Schmitz R. J., Ackerman T. A., Schulz M. R. (2011). Lower body stiffness and muscle activity differences between female dancers and basketball players during drop jumps. *Sports Health*.

[43] Simons C., Bradshaw E. J. (2016). Do accelerometers mounted on the back provide a good estimate of impact loads in jumping and landing tasks?. *Sports Biomechanics*.

[44] Staniak Z., Buśko K., Górski M., Pastuszak A. (2016). Accelerometer profile of motion of the pelvic girdle in breaststroke swimming. *Journal of Human Kinetics*.

